# The mechanotransduction-immune axis in organ fibrosis: dual regulatory mechanisms and translational therapeutic perspectives

**DOI:** 10.3389/fimmu.2025.1670213

**Published:** 2025-10-24

**Authors:** Chao Lei, Dongjie Wu, Houyan Zhang, Zhiya Yang, Bohao Huang, Jie Wang, Yanbo Li, Wenliang Lv

**Affiliations:** ^1^ Department of Infection, Guang’anmen Hospital, China Academy of Chinese Medical Sciences, Beijing, China; ^2^ Department of Oncology, Wangjing Hospital, Chinese Academy of Traditional Chinese Medicine, Beijing, China; ^3^ School of Traditional Chinese Medicine, Beijing University of Traditional Chinese Medicine, Beijing, China

**Keywords:** organ fibrosis, mechanotransduction, immune, extracellular matrix, therapeutic target

## Abstract

Organ fibrosis represents a final common pathway of chronic tissue injury, characterized by persistent extracellular matrix (ECM) accumulation and progressive loss of organ function. While canonical inflammatory and profibrotic cascades have been extensively studied, emerging evidence highlights the pivotal role of mechanotransduction-the process by which cells sense and transduce biomechanical cues-in orchestrating immune responses and driving fibrotic remodeling. This review conceptualizes the mechanotransduction-immune axis as a dual regulatory network wherein mechanical forces not only activate profibrotic signaling in resident stromal cells but also dynamically reprogram immune cell phenotypes and functions. We systematically delineate the molecular and cellular mechanisms by which matrix stiffness, shear stress, and mechanical stretch engage integrins, focal adhesion kinase, Piezo1, and TRPV4 to coordinate inflammatory signaling and ECM remodeling. Additionally, we discuss how immune cells, including macrophages, T cells, and neutrophils, sense and respond to mechanical inputs to amplify profibrotic responses. Finally, we summarize emerging translational therapeutic perspectives targeting this mechanotransduction-immune interplay, encompassing small-molecule inhibitors, nanomedicine approaches, gene editing technologies, and cell therapies. By integrating mechanistic insights and translational strategies, this review aims to provide a comprehensive framework for understanding and therapeutically targeting the mechanotransduction-immune axis in organ fibrosis.

## Highlights

- Matrix stiffness, shear stress, and tensile forces activate key profibrotic mechanosensors.
- The mechanotransduction-immune axis forms an integrated regulatory network in fibrosis.
- Immune-mechanical crosstalk amplifies profibrotic signaling in organ fibrosis.
- Emerging therapeutic strategies and smart nanocarriers targeting this axis offer promising precision interventions for fibrosis across organs.

## 1 Introduction

Organ fibrosis represents a core pathological process in end-stage organ failure across multiple chronic diseases, commonly affecting vital organs such as the heart, liver, lungs, and kidneys. It is primarily characterized by excessive deposition and aberrant remodeling of the extracellular matrix (ECM), ultimately leading to loss of tissue elasticity and progressive functional decline (1, 2). In recent years, with in-depth investigation into fibrogenesis, the mechanical properties of tissues have emerged as a critical focus in fibrosis research. Mechanotransduction refers to the biological process wherein cells convert external mechanical stimuli-including matrix stiffness, fluid shear stress, and tensile forces-into biochemical signals. This conversion activates specific signaling pathways, thereby modulating cellular proliferation, migration, polarization, and gene expression (3). The pivotal role of mechanical stimuli in fibrosis progression is widely recognized, and their function in regulating inflammation and immune responses is increasingly emphasized (4). Under pathological conditions, significant alterations in tissue biomechanics occur. Concurrently, the structure and function of mechanotransduction molecules at the cellular level-such as adhesion molecules, ion channels, and cytoskeletal proteins-are modified, thereby influencing the activity of multiple associated signaling pathways. Such biomechanical dysregulation manifests across fibrotic processes in various organs. Myofibroblasts serve as the primary ECM producers, and their activation constitutes a critical step in fibrosis development. However, this process does not occur in isolation. The immune system, particularly innate and adaptive immune cells, plays an indispensable role in regulating myofibroblast activation and fibrotic responses (5).

In recent years, with the advancement of the emerging interdisciplinary field of mechanoimmunology, it has become increasingly recognized that mechanical signals not only play crucial roles in embryonic development, cardiovascular homeostasis, and bone metabolism but also profoundly participate in inflammation and immune regulation (6). During circulation, immune cells are continually exposed to diverse mechanical environments, modulated by forces such as stretch, shear, and compression. Concurrently, tissue-resident immune cells persistently experience mechanical stimuli from their local microenvironment. Furthermore, the structure and spatial architecture of the ECM constitute complex mechanical inputs that further influence immune cell functional states. These mechanical signals are transmitted through mechanosensors on the cell membrane and intracellularly, integrated into mechanotransduction networks, and subsequently activate multiple signaling pathways. Aberrant mechanical environments are considered key triggers for dysregulated interplay between structural cells and immune cells. This dual-dimensional regulatory mechanism-simultaneously involving both mechanical and immunological aspects-represents the core focus of mechanoimmunology research.

**Figure 1:**
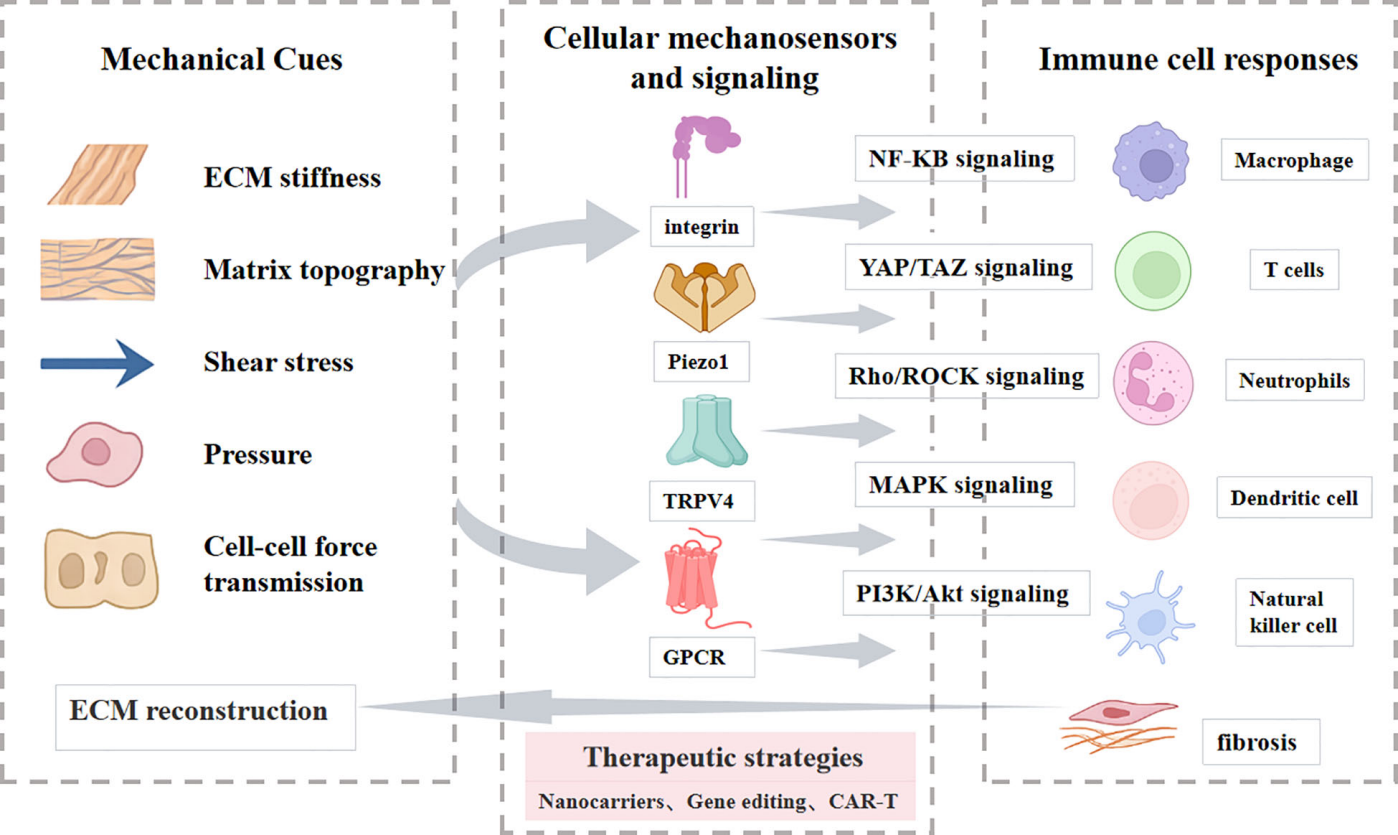
Overview of the review structure. A schematic illustration guiding readers through the main sections and topics covered in this review.

This review will systematically delineate the driving mechanisms of mechanical signals in fibrosis progression and their regulatory effects on immune cell behavior. We will specifically emphasize the response patterns of different immune cells to mechanical stimuli and their functional roles within the fibrotic microenvironment. By integrating current research advances, we aim to provide novel perspectives for understanding the impact of the mechanical microenvironment on immune regulation and fibrotic pathology, thereby establishing a theoretical framework for developing relevant therapeutic strategies (**Figure 1**).

## 2 Mechanosensation and fibrosis-driving mechanisms

Mechanotransduction converts external physical stimuli into intracellular signaling events, ultimately inducing alterations in cellular behaviors such as proliferation, migration, gene expression regulation, and differentiation (7, 8). Cellular responses to mechanical stimuli rely on mechanosensors distributed across the extracellular space, cytoplasm, and nucleus to perceive and integrate mechanical cues, thereby activating specific biological reactions. Key mechanosensors identified to date include Integrins, FAK, Mechanosensitive ion channels, Proteoglycan complexes, Cytoskeletal components, and Nuclear structural proteins (9–12). These sensors detect extracellular mechanical changes and transduce them into biochemical signals through multiple downstream pathways, including ROCK, YAP/TAZ, MAPK, PI3K/Akt pathway, and NF-κB (13–15).

### 2.1 ECM stiffness and topography sensing

#### 2.1.1 Matrix stiffness

In healthy tissues, the ECM maintains moderate elasticity and organized fiber alignment. Fibrotic remodeling leads to excessive deposition of collagen and other matrix components, markedly increasing stiffness and altering microarchitecture (9, 16). Cells sense increased rigidity mainly via integrins, which cluster upon ligand engagement and recruit focal adhesion kinase FAK and Src family kinases (17–20). This activation drives RhoA/ROCK signaling, promoting stress fiber assembly and reinforcing cytoskeletal tension. Elevated intracellular force facilitates the nuclear translocation of mechanosensitive transcription factors, which upregulate profibrotic genes including ACTA2 and COL1A1. Concurrently, matrix stiffening promotes the release and activation of latent TGF-β, which synergizes with integrin signaling to amplify Smad-dependent transcriptional programs (21–23). For example, in hepatic stellate cells, stiff substrates enhance FAK–YAP cooperation, sustaining ECM production and myofibroblast activation (24, 25). These events illustrate how matrix stiffening not only drives intracellular mechanotransduction but also establishes a vicious cycle in which integrin–FAK–ROCK and YAP/TAZ–Smad pathways reinforce each other. This positive feedback perpetuates fibroblast activation and excessive ECM deposition, thereby locking tissues into a progressively fibrotic state.

#### 2.1.2 Matrix topography

Matrix topography refers to nanoscale and microscale features such as fiber alignment, roughness, and porosity. In fibrosis, highly oriented collagen bundles guide fibroblast migration along fiber axes, reinforce directional traction forces, and further remodel ECM architecture. Nanoscale surface roughness alters membrane curvature and activates mechanosensitive ion channels, including Piezo1 and TRPV4, triggering calcium influx and downstream calcineurin-NFAT signaling (26–29). These pathways cooperate with YAP/TAZ to drive contractile differentiation. Surface roughness also modulates integrin clustering and focal adhesion distribution (30), enhancing fibroblast contractility and α-SMA expression. Additionally, reduced matrix porosity constrains cell spreading, increases cytoskeletal tension, and promotes YAP nuclear localization, while simultaneously limiting immune cell infiltration and facilitating local accumulation of profibrotic mediators (31, 32). These micro- and nanoscale topographical changes couple structural remodeling of the ECM to intracellular mechanotransduction. By synchronously regulating fibroblast activation and immune cell accessibility, altered topography reinforces ECM deposition and sustains a microenvironment conducive to progressive fibrosis.

Taken together, cell–matrix interactions transform extracellular stiffness or architecture into intracellular signaling events that activate fibroblasts and immune cells. Through integrin, FAK/Src, and downstream RhoA–ROCK and MAPK cascades, these signals converge on enhanced cytoskeletal contractility and YAP/TAZ-dependent transcription. The outcome is excessive ECM production, which in turn stiffens the matrix and perpetuates a profibrotic feedback loop.

### 2.2 Shear stress sensing

Shear stress is one of the primary fluid mechanical forces that shape cellular responses and tissue remodeling (33). Cells perceive shear forces through various membrane-associated structures, including primary cilia, microvilli, the glycocalyx, intercellular junctions, integrins, GPCRs, and mechanosensitive ion channels (34, 35). These structures transduce mechanical cues by physical deformation, tension shifts, or conformational activation, initiating mechanochemical signaling cascades (36, 37).

The primary cilium, a microtubule-based organelle, is a critical shear sensor. Under flow, its basal body and associated ion channels trigger calcium influx and downstream signaling (3, 34, 38). Microvilli and the glycocalyx increase the effective surface area for shear force capture and maintain barrier function, particularly in endothelial cells (35, 39). Integrins sense shear via focal adhesion complexes, activating FAK-Src and RhoA pathways to regulate cytoskeletal tension (40–42). GPCRs respond to membrane deformation through conformational shifts that initiate G protein–mediated signaling cascades. Recent studies have identified OXGR1, a GPCR responsive to oxoglutarate, as a critical regulator of fibroblast function. Upon activation, OXGR1 engages the PI3K/Akt pathway, thereby promoting fibroblast proliferation and contributing to extracellular matrix remodeling. This mechanistic link highlights the role of GPCR-mediated PI3K/Akt activation in the pathogenesis of fibrotic tissue dynamics (43, 44). Piezo1 detects shear stress to mediate calcium entry and regulate vascular tone, while TRPV4 contributes to volume regulation and pro-inflammatory amplification, aberrant activation of these channels is implicated in fibrosis across organs (45). Collectively, these diverse shear-sensing mechanisms converge on PI3K/Akt, RhoA–ROCK, and NF-κB pathways, orchestrating fibroblast activation, immune modulation, and ECM remodeling. By integrating mechanical flow cues into cellular signaling, shear stress acts as a central driver of the self-reinforcing cycle that underlies chronic fibrotic progression.

### 2.3 Pressure sensing

In contrast, pressure exerts perpendicular forces on cells, including hydrostatic pressure, trans-epithelial or trans-endothelial gradients, and interstitial fluid pressure (IFP). Pressure alters cell morphology and tension states, activating mechanosensors and downstream pathways. Abnormal pressure contributes to perfusion deficits, apoptosis, autophagy, and inflammatory mediator release in hepatic (46), pulmonary (47), renal (48), and scleral fibrosis (49).

Hydrostatic pressure, common in perfused tissues, increases with fluid accumulation and transmits load through sinusoids or interstitial spaces. For example, portal hypertension in liver fibrosis impairs liver sinusoidal endothelial cells (LSEC) barrier function and activates hepatic stellate cells (HSCs) mechanically (46, 50). Hydrostatic pressure enhances cytoskeletal remodeling and fibrotic gene expression via RhoA/ROCK and YAP/TAZ pathways, exacerbating tissue stiffening and fibrogenesis (51).

Trans-epithelial and trans-endothelial gradients are prevalent in renal tubules, alveoli, and bile ducts. Elevated gradients increase mechanical load, trigger stress responses, and disrupt barriers. In kidney injury, intraluminal pressure activates Piezo1, leading to calcium influx, EMT induction, and collagen deposition (52).

IFP is persistently elevated in fibrotic tissues due to matrix accumulation, crosslinking, and lymphatic dysfunction, forming an “interstitial pressure trap.” This environment upregulates hypoxia-inducible factor-1α (HIF-1α), vascular endothelial growth factor (VEGF), and matrix metalloproteinases (MMPs), altering metabolism and oxidative stress (53–55). Sustained compression reorganizes F-actin and promotes YAP/TAZ and MRTF-A nuclear translocation, activating TGF-β autocrine signaling and Smad-dependent transcription to establish profibrotic feedback loops (49, 56). These distinct forms of pressure sensing converge on cytoskeletal remodeling, mechanosensitive ion channel activation, and YAP/TAZ–Smad signaling. By coordinating fibroblast activation, barrier dysfunction, and metabolic reprogramming, pressure not only reflects tissue injury but also acts as a potent driver of the self-reinforcing cycle that sustains fibrosis progression.

In summary, shear stress and interstitial flow are sensed by mechanosensitive channels such as Piezo1 and TRPV4, which activate calcium influx, NF-κB, and PI3K/Akt signaling. These events shape macrophage polarization and endothelial activation, thereby amplifying local inflammation and fibroblast activation. Ultimately, fluid mechanical cues reinforce ECM deposition and accelerate fibrotic progression.

### 2.4 Cell-cell mechanical coupling

#### 2.4.1 Cadherin-mediated mechanotransduction

Beyond cell-matrix interactions, direct cell-cell mechanical communication, primarily mediated by cadherins, plays a critical role in the pathophysiology of organ fibrosis. Cadherins are calcium-dependent transmembrane glycoproteins that function as dynamic mechanosensing and signal transduction hubs, converting physical forces into biochemical signals (57, 58). The core mechanism involves force-dependent conformational changes that enhance trans-cellular binding and allosterically propagate tension intracellularly, stabilizing β-catenin and recruiting vinculin to bridge the cadherin-catenin complex to the actin cytoskeleton (59–62). This reinforced adhesion platform recruits and activates FAK and Src kinases, initiating downstream RhoA/ROCK signaling to amplify actomyosin contractility and regulate the nucleocytoplasmic shuttling of YAP/TAZ (13).

Emerging evidence positions cadherin-mediated coupling, particularly via Cadherin-11, as a pivotal mechano-immune bridge in fibrosis. A seminal study demonstrated that Cadherin-11 facilitates robust adhesion between M2-like macrophages and myofibroblasts, creating a niche enriched with active TGF-β (3, 57, 63, 64). This heterotypic adhesion not only enables macrophages to mechanically activate fibroblasts but also potentially reinforces the pro-fibrotic polarization of macrophages themselves through sustained mechanical feedback. Further illuminating this paradigm, a recent study by Astrab et al. reveals that Cadherin-11 directly interacts with the mechanosensor Piezo1 and cooperates with IL-6 signaling to drive fibroblast activation (65). This emerging model posits Cadherin-11 as a signaling node that integrates mechanical stress perception (via Piezo1) with inflammatory cues to amplify pro-fibrotic responses.

In summary, cadherin-mediated mechanotransduction provides a fundamental pathway for bi-directional mechanical and chemical crosstalk between stromal and immune cells. Its role as a mechano-immune bridge offers a compelling molecular framework for understanding how physical cues sculpt the immune landscape in fibrosis, presenting a promising target for dual-mechanism therapeutic interventions.

#### 2.4.2 Paratensile signaling

Paratensile signaling represents a sophisticated mode of long-range mechanical communication in fibrosis, operating through three sequential phases: force generation, propagation, and cellular response (66, 67). The process initiates with force generation by activated myofibroblasts, which exert substantial contractile forces on the ECM via integrin-mediated focal adhesions. These forces are propagated through stiffened and aligned collagen bundles, which serve as conduits for tensile stress over distances exceeding several cell lengths. The efficiency of this transmission depends critically on collagen cross-linking by enzymes such as LOX (1, 68). Distal cells then sense these mechanical cues via mechanosensors like Piezo1 or discoidin domain receptor 2 (DDR2), activating pro-fibrotic transcriptional programs.

Experimental models have provided direct evidence for paratensile signaling. For instance, *in vitro* studies using atomic force microscopy (AFM) to apply localized force via collagen fibers have shown that remotely located fibroblasts upregulate pro-fibrotic genes—a response abolished upon inhibition of Piezo1 or DDR2 (66). Ex vivo models of the fibrotic–nonfibrotic interface further demonstrate that mechanical force propagation through collagen networks is necessary for the expansion of fibrotic lesions. Laser ablation of intervening collagen fibers between activated myofibroblasts and quiescent fibroblasts abrogates this activation, confirming the ECM’s role as a mechanical conduit (67, 69).

For example, in liver fibrosis, portal hypertension and inflammation remodel liver sinusoidal endothelial cells and enhance their attachment to perisinusoidal collagen. Tensile forces transmitted along collagen bundles mechanically activate hepatic stellate cells, increasing profibrotic gene expression via integrin-FAK-YAP/TAZ pathways (50, 70). Thus, paratensile signaling establishes the remodeled ECM as a long-range communication network that drives fibrosis progression by enabling localized mechanical activation to spread radially beyond injury sites (71). This concept expands our understanding of stromal–mechanical crosstalk and suggests new therapeutic strategies aimed at disrupting pathological force transmission, such as targeting LOX or Piezo1.

#### 2.4.3 Cell-matrix-cell interactions

A further level of coupling arises from cell–matrix–cell interactions. Here, cells remodel their local ECM while transmitting mechanical signals to other cells anchored within the same matrix. In pulmonary fibrosis, fibroblast-generated traction reorganizes and stiffens collagen networks, enhancing integrin clustering and focal adhesion maturation in adjacent fibroblasts. This mechanical relay synchronizes myofibroblast activation and contractile differentiation across tissues (32, 72). Similarly, in cardiac fibrosis, fibroblast tension increases regional matrix stiffness and promotes sustained YAP/TAZ activation, establishing a feedforward loop of fibrosis progression (73).

**Figure 2:**
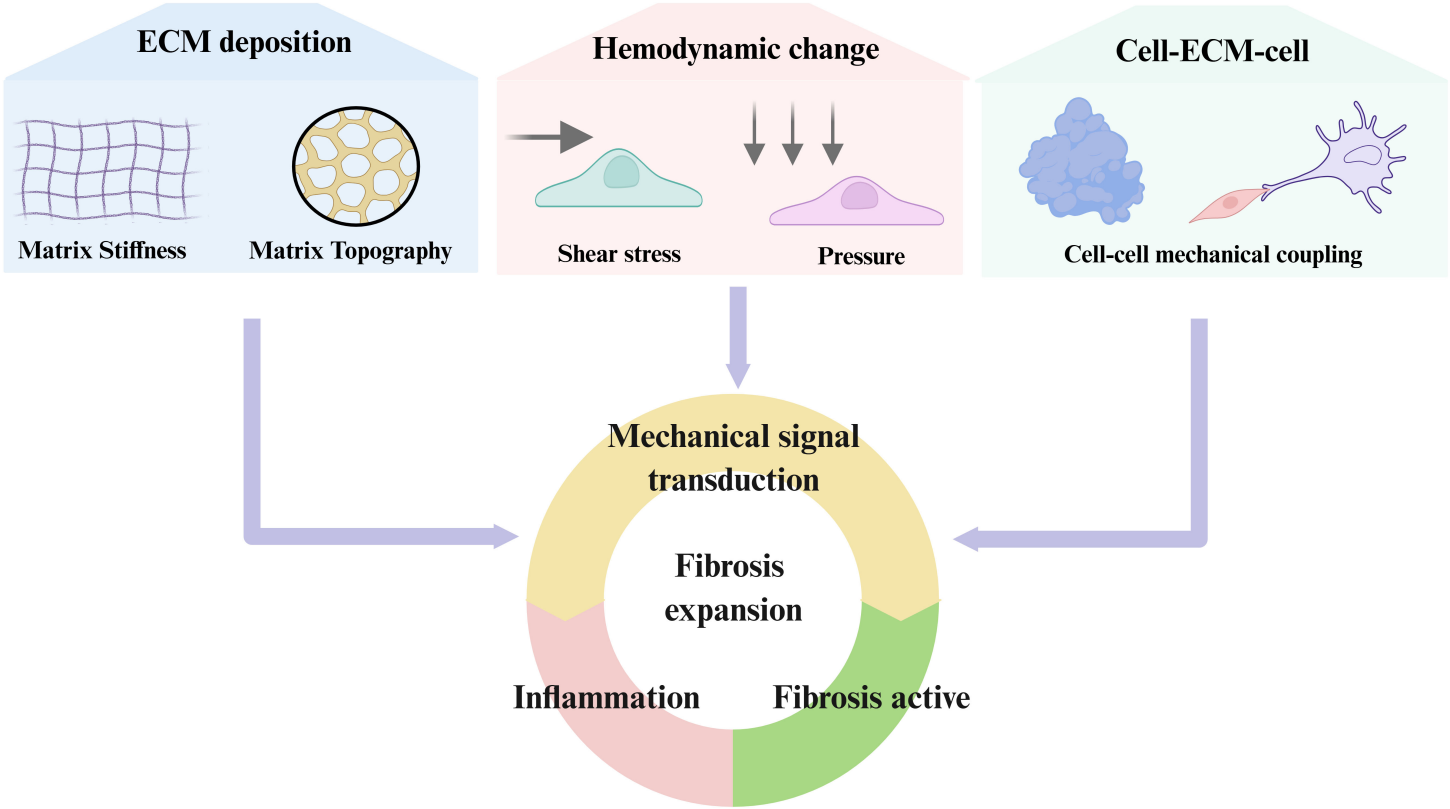
Principal biomechanical cues driving fibrotic activation. Fibrosis is orchestrated by dynamic changes in extracellular matrix stiffness, matrix topography, aberrant hemodynamics (shear and pressure), and mechanical crosstalk between cells and the ECM. These biomechanical cues are sensed through integrins, cadherins, GPCRs, mechanosensitive ion channels, and cytoskeletal linkers, which activate downstream signaling cascades including RhoA/ROCK, YAP/TAZ, MAPK, PI3K/Akt, and NF-κB. Collectively, these pathways converge on fibroblast activation, immune modulation, and excessive ECM deposition, thereby establishing self-reinforcing feedback loops that drive and sustain progressive fibrosis.

**Figure 3:**
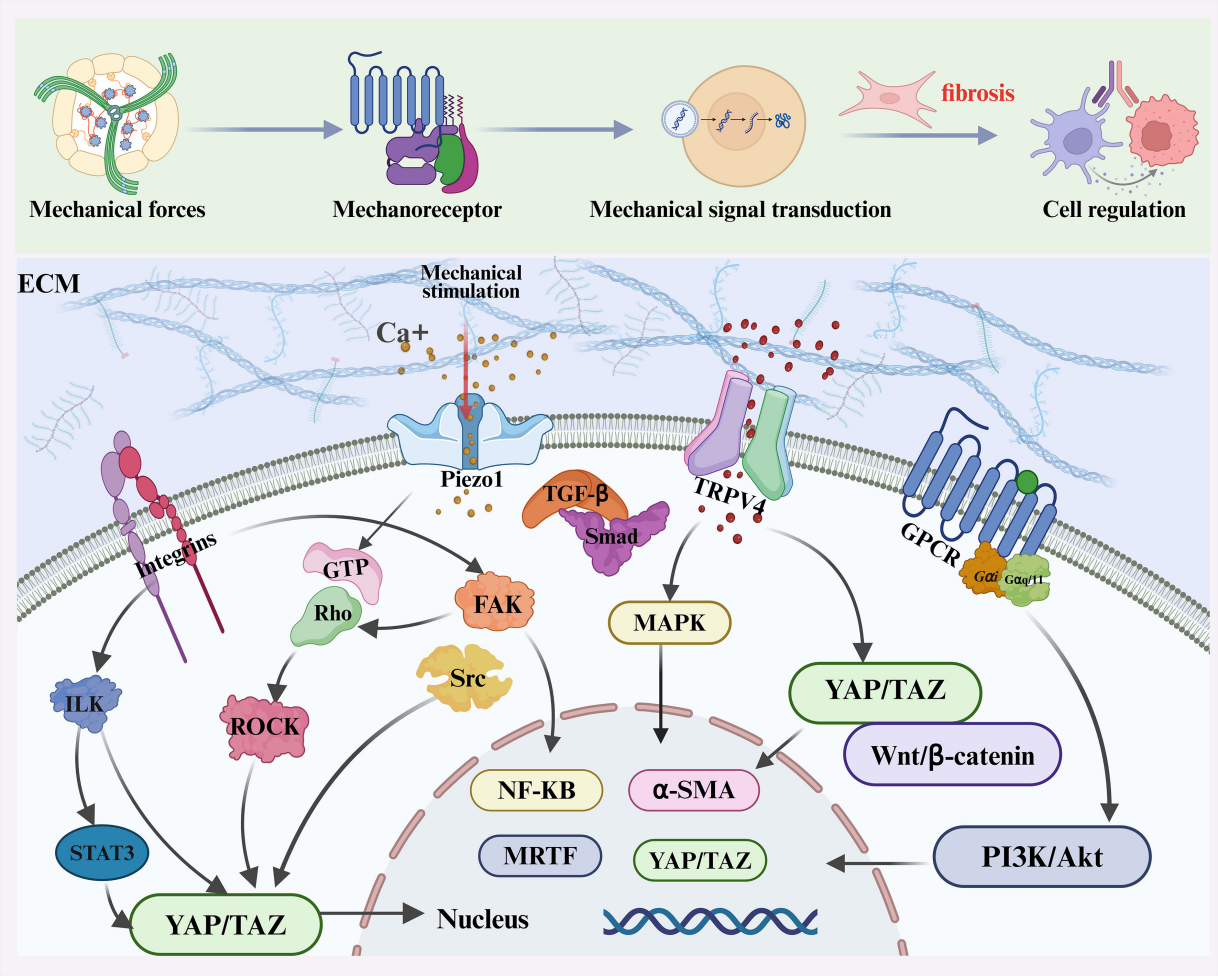
Overview of mechanotransduction and immune regulation in fibrosis. Mechanical forces—including stiffness, shear stress, pressure, stretch, and topography—are sensed by mechanoreceptors such as Integrins, Piezo1, TRPV4, and G protein–coupled receptors (GPCRs). These receptors activate downstream signaling cascades including focal adhesion kinase (FAK)/Src, Rho/ROCK, MAPK, PI3K/Akt, TGF-β/Smad, and YAP/TAZ pathways, promoting cytoskeletal remodeling, profibrotic gene expression, and ECM deposition.

Overall, force transmission between neighboring cells through adherens junctions, gap junctions, and cytoskeletal connections creates a multicellular amplification system. Mechanical forces propagate from fibroblasts to immune cells and back, sustaining ROCK, YAP/TAZ, and NF-κB activation across cell populations. This cross-talk expands fibrotic foci and drives the transition from local injury to widespread fibrosis. While the previous sections focused on how mechanical cues are sensed and transmitted at the cellular level, the next sections will dissect the regulation of immune cells in the mechano-fibrotic axis. **Figure 2** and **Figure 3** provide an integrated overview of the major mechanical cues and mechanotransduction pathways involved in fibrosis progression.

## 3 Regulation of immune cells in the mechano-fibrotic axis

Notably, mechanically driven remodeling of the extracellular matrix also creates a mechanically adaptive microenvironment for immune cells. Immune cells are not only key regulators of inflammation and tissue repair but also highly sensitive to changes in the mechanical microenvironment. This mechanosensitivity enables them to sense variations in matrix stiffness, fluid shear stress, and intercellular tension, thereby modulating their migration, activation, secretory profile, and differentiation. In this context, immune cells act as both responders and amplifiers of mechanical signals.

### 3.1 Macrophage Mechanosensing and Polarization

Macrophage functional plasticity, encompassing their capacity to polarize into pro-inflammatory (M1) or pro-repair/pro-fibrotic (M2) states, is critically implicated in fibrosis pathogenesis. Beyond biochemical cues, the biomechanical properties of the fibrotic microenvironment—notably increased matrix stiffness, fluid shear stress, and intercellular tension—are now recognized as active instructors of macrophage polarization (6, 74, 75). This mechanical instruction is decoded via a sophisticated mechanotransduction network that converts physical forces into biochemical signals, ultimately reprogramming macrophage gene expression and function (76). The core mechanosensing apparatus in macrophages comprises integrin-based focal adhesions and mechanosensitive ion channels. Upon adhesion to pathologically stiff matrices, integrin clustering activates FAK, initiating downstream RhoA/ROCK signaling to enhance actomyosin contractility and cytoskeletal tension (17, 77). Concurrently, mechanical stimuli directly activate channels such as Piezo1 and TRPV4, inducing rapid Ca^2+^ influx that serves as a pivotal second messenger (78, 79).

These proximal sensing events converge on key signaling hubs that cooperatively dictate polarization fate. The transcriptional co-activators YAP/TAZ are central mediators. Under high cytoskeletal tension, inhibition of the LATS1/2 kinases in the Hippo pathway permits YAP/TAZ nuclear translocation. There, they partner with transcription factors like TEAD to drive pro-fibrotic (e.g., CTGF, CYR61) and pro-inflammatory gene expression (13, 80). Stiffness-driven M1 polarization is facilitated by the Piezo1-YAP axis, while YAP/TAZ inhibition attenuates this inflammatory response (81). Complementarily, NF-κB signaling is potently activated by mechanical cues; Piezo1-mediated Ca^2+^ influx can engage the calcineurin-NFAT pathway, synergizing with canonical IKK-NF-κB signaling to upregulate classic M1 markers like TNF-α and IL-1β (81, 82).

As fibrosis progresses, sustained mechanical stimulation can promote a shift toward an M2 phenotype. Persistent RhoA/ROCK signaling on stiff substrates not only maintains YAP/TAZ activation but also cooperates with cytokine-activated STAT6 to induce expression of M2 markers (e.g., Arg1, CD206) and enhance secretion of TGF-β1 (78, 82, 83). This M2-polarized state, in turn, activates fibroblasts via paracrine signaling, exacerbating ECM deposition and matrix stiffness—thereby establishing a self-perpetuating “mechano-immune positive feedback loop”. Notably, these mechanically induced phenotypic shifts can be stabilized through epigenetic modifications, conferring a “mechanical memory” that persists even after the initial mechanical insult is removed (84).

In summary, the M1/M2 transition in macrophages is precisely orchestrated by an integrated mechanotransduction network. This network employs integrins/FAK and Piezo1/TRPV4 as primary sensors, with YAP/TAZ, NF-κB, and ROCK serving as core signaling axes to translate biomechanical cues into transcriptional programs that guide immune responses. A deeper dissection of this mechanism is crucial for developing novel therapies targeting the mechano-immune axis in fibrosis.

### 3.2 T cells mechanotransduction

T cell mechanosensing primarily occurs via T cell receptor (TCR) engagement, integrin-mediated adhesion, and the cytoskeletal machinery. Stiff matrices activate ILK–STAT3 and YAP/TAZ signaling, enhancing RORγt expression and promoting Th17 differentiation. Th17 cells secrete IL-17A, IL-22, and GM-CSF, which induce EMT, fibroblast activation, and inflammatory loops (85–87). Matrix viscoelasticity modulates AP-1 protein expression, generating distinct T cell subsets (88). In addition, shear stress induces sustained T cell activation and reprogramming through Piezo1-mediated calcium influx, which in turn enhances downstream signaling pathways such as NF-κB and NFAT (4). Moderate shear also increases major histocompatibility complex (MHC)-I and CD86 expression and cytokine secretion, enhancing T cell activation capacity (89, 90).

In contrast, Tregs are central to maintaining homeostasis and suppressing inflammation, but their stability is also mechanically regulated. Soft, low-tension environments support Foxp3 expression and suppressive function. In stiffened matrices, YAP/TAZ activation downregulates Foxp3, driving Treg conversion toward Th17/Th1 phenotypes and loss of immunoregulatory capacity (91, 92). In liver fibrosis models, Treg instability often coexists with Th17 expansion, suggesting that mechanical disturbance underlies (93, 94).

### 3.3 Neutrophils mechanotransduction

Neutrophils are highly responsive to mechanical cues, particularly matrix stiffness and viscoelasticity. Stiff substrates promote increased neutrophil spreading, ROS production, and the release of neutrophil extracellular traps (NETs), a process termed NETosis (95, 96). In stiff or stretched microenvironments, neutrophils are prone to NETosis, releasing DNA webs enriched in tissue factor (TF), histones, myeloperoxidase (MPO), and neutrophil elastase (97–99). In fibrotic tissues, excessive NET formation has been implicated in sustaining chronic inflammation and stimulating profibrotic. For example, in murine models, NET inhibition reduces fibroblast activation and collagen accumulation, validating their pathological role (100).

Mechanosensitive ion channels such as Piezo1 and TRPV4 also contribute to T cell and neutrophil responses by regulating intracellular calcium dynamics and cytoskeletal remodeling (98, 101–103). Although most studies have focused on acute inflammatory contexts, these pathways are likely relevant to chronic fibrotic settings, where persistent mechanical remodeling creates proinflammatory microenvironments.

### 3.4 Dendritic cells mechanotransduction

Dendritic cells (DCs) are highly sensitive to mechanical cues in their microenvironment, including matrix stiffness, viscoelasticity, and interstitial flow (104). Increased substrate stiffness promotes DC spreading, actin cytoskeletal reorganization, and enhanced maturation, characterized by upregulated expression of MHC-II, CD80, and CD86, which facilitates T cell priming (105, 106). Mechanical forces also influence DC cytokine secretion: stiffer matrices enhance the production of IL-6, TNF-α, and TGF-β, potentially contributing to fibroblast activation and extracellular matrix deposition (105). Mechanosensitive pathways, such as Piezo1-mediated Ca^2+^ influx, regulate DC motility and antigen-presenting functions, allowing DCs to act as both sensors and modulators of mechanically altered fibrotic microenvironments (107, 108). DCs translate mechanical perturbations into immunomodulatory signals that can either exacerbate or resolve fibrosis, positioning them as critical mechano-immune hubs in fibrotic progression and potential therapeutic targets.

### 3.5 Natural killer cells mechanotransduction

Natural killer (NK) cells, crucial effectors of innate immunity, are increasingly recognized as responsive mechanosensors whose cytotoxic and migratory functions are tuned by mechanical cues within the tissue microenvironment (6). In fibrotic contexts, pathologically stiffened ECM enhances NK cell cytotoxicity, a process mediated significantly by the mechanosensitive ion channel Piezo1. Piezo1 activation facilitates calcium influx, promoting polarization of the microtubule-organizing center and directed release of cytotoxic granules toward target cells (109). Beyond static stiffness, fluid shear stress dynamically regulates NK cell function. Notably, the activating receptor NKG2D has been implicated in mechanosensation. Under physiological shear stress, ligand engagement induces conformational changes in NKG2D, triggering downstream signaling involving VAV1 and PI3K phosphorylation, which specifically enhances granzyme B delivery to target cells without broadly upregulating cytokine secretion (110).

**Figure 4:**
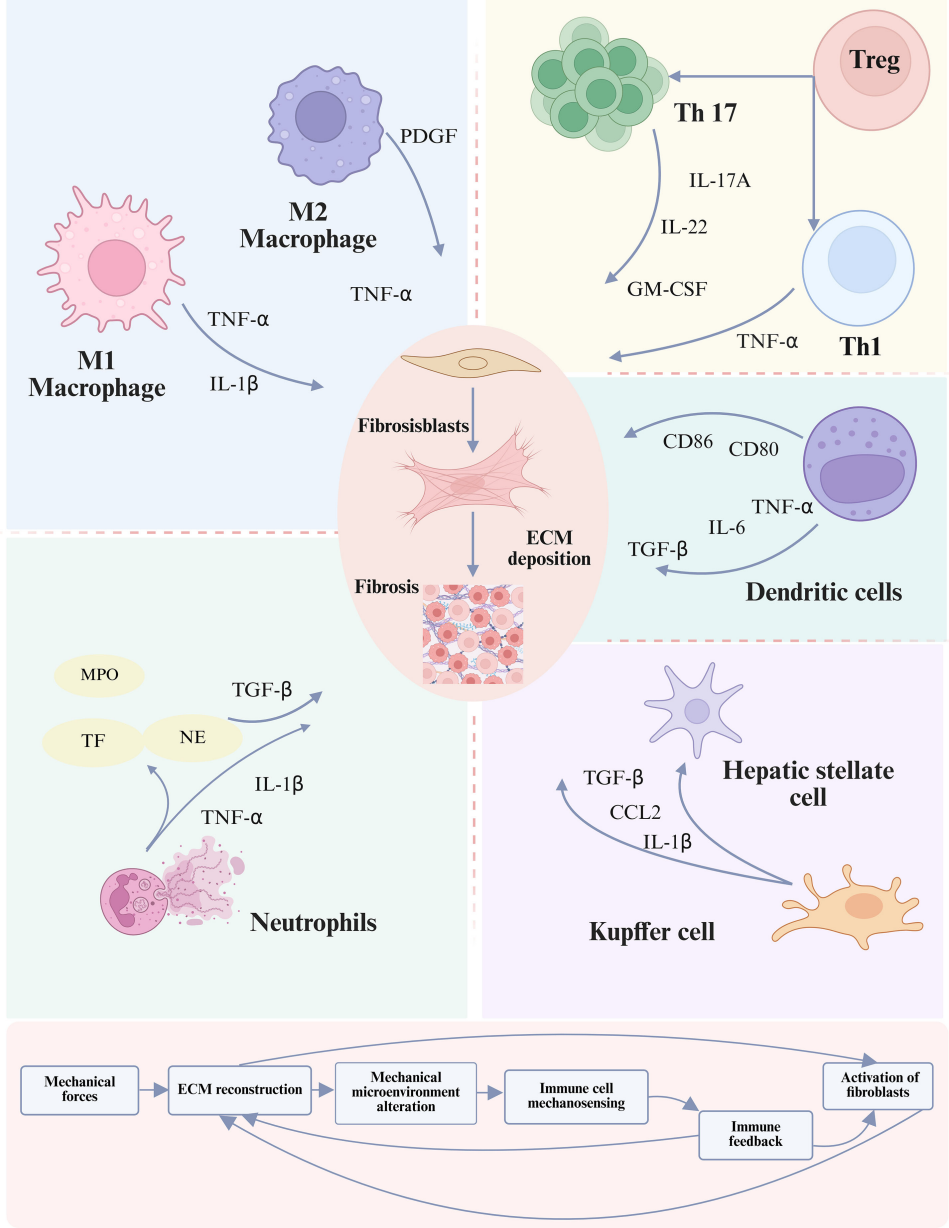
Role of the immune microenvironment in fibrosis progression. Immune cells both contribute to and regulate fibrotic remodeling by modulating the activation of fibroblasts and HSCs. This regulation occurs directly through cell-cell contact or indirectly via the release of cytokines, chemokines, and ligand-receptor signaling, thereby shaping the trajectory of extracellular matrix deposition and tissue scarring (113). The lower panel illustrates a positive feedback loop in which activated fibroblasts not only produce excessive ECM but also stimulate the mechanotransduction–immune axis, thereby perpetuating fibrosis progression through iterative interactions between mechanical cues and immune responses (105). To date no definitive evidence shows that mechanical cues such as ECM stiffness directly switch NK cells to a pro-fibrotic mode, representing a gap in the mechanotransduction-immune axis understanding.

Within the fibrotic niche, characterized by progressive matrix stiffening and altered interstitial flow, the functional impact of these mechanical pathways on NK cells remains an open question. While direct evidence for a mechanically-driven pro-fibrotic NK phenotype is scarce, it is plausible that chronic mechanical stimulation could modulate their activity, potentially shifting the balance between cytotoxic and immunomodulatory functions (6, 111). In summary, NK cells are active participants in mechano-immunological crosstalk (112). Understanding how Piezo1 and NKG2D integrate mechanical signals in the context of fibrotic architecture is essential to fully delineate their role in the mechano-immune axis of organ fibrosis. **Figure 4** demonstrates that immune cell subsets contribute to fibrosis by releasing pro-inflammatory cytokines and engaging in direct or paracrine interactions with fibroblasts.

### 3.6 Immune cell feedback loops reinforcing fibrotic mechanotransduction

#### 3.6.1 Immune cell-extracellular matrix feedback amplifies mechanical signal transduction

Immune cells not only passively respond to changes in the mechanical microenvironment but also actively remodel the mechanical properties of the ECM, forming an “immune–matrix–mechanical” closed feedback loop that drives fibrosis progression. As local ECM stiffness increases, immune cells undergo adaptive remodeling at multiple levels-including morphology, adhesion molecule expression, and activation of mechanosensitive pathways-to enhance their mechanical responsiveness. For example, in high-stiffness microenvironments, classically activated M1 macrophages significantly upregulate integrin αMβ2 (CD11b/CD18), strengthening their adhesion to ECM components such as type I collagen and fibronectin. This high-affinity binding activates the integrin-FAK-ROCK signaling axis, promoting actomyosin contractility. The resulting active traction forces are transmitted into the surrounding matrix, stimulating adjacent fibroblasts through YAP/TAZ and RhoA signaling to adopt a myofibroblastic phenotype (114, 115). Activated macrophages also modulate ECM mechanics through secreted factors. M2 macrophages secrete TGF-β1, which can activate fibroblasts to produce LOX, thereby promoting collagen crosslinking and matrix stiffening (68). This chemical-mechanical coupling drives early matrix soft-to-stiff transitions and maintains high matrix rigidity during advanced fibrosis. Importantly, the mechanical signals generated by immune cells reinforce their own profibrotic polarization. In stiff matrices, macrophages sustain M2 phenotypes and secretion of TGF-β1 and PDGF via persistent ROCK2 activation, creating a positive feedback loop that amplifies mechanoinflammatory signaling (116). In animal models of pulmonary fibrosis, macrophage-specific ROCK2 inhibition reduces contractile force generation, suppresses profibrotic signaling, attenuates fibroblast activation (83).

#### 3.6.2 Immune cells establish force-transmitting networks via cell-cell adhesion

Immune cells propagate mechanical signals through direct physical contacts, forming structural networks that coordinate mechanotransduction across tissues. Adhesion molecules form the basis of these “force transmission networks,” simultaneously mediating biochemical signaling and coordinating intercellular mechanical stress. A classical example is LFA-1 (CD11a/CD18) binding to ICAM-1. LFA-1–ICAM-1 interactions stabilize immune synapses under shear stress (117, 118), supporting persistent immune activation in fibrotic lungs (119, 120). Beyond classical synapses, heterotypic mechanical contacts between immune and stromal cells are critical in fibrotic disease. In lung, liver, and kidney fibrosis, M2 macrophages form stable adhesions with activated fibroblasts via N-cadherin–β-catenin complexes, enabling bidirectional mechanical signaling that promotes myofibroblast differentiation and matrix remodeling (59, 60, 65).

### 3.7 Epigenetic regulation in the mechano-immune axis of fibrosis

Emerging evidence underscores that epigenetic regulation serves as a critical molecular interface translating transient mechanical cues into sustained pro-fibrotic phenotypes, thereby establishing a “mechanical memory” that perpetuates fibrosis even after the initial insult is resolved (121). This mechano-epigenetic axis operates as a self-reinforcing loop across both stromal and immune cells, constituting the core of the mechano-immune axis.

The process is initiated by mechanical force sensing through integrins and ion channels (e.g., Piezo1), leading to the activation of downstream effectors such as YAP/TAZ and MRTF in stromal cells. These transcriptional co-activators recruit chromatin-modifying complexes to profibrotic gene loci (122). A key mechanistic insight comes from the recently identified EZH2–YAP feedback loop. In fibrotic kidneys, upregulated EZH2—a histone methyltransferase—deposits the repressive mark H3K27me3 at the promoter of LATS1, a core inhibitor of the Hippo pathway. This epigenetic silencing promotes YAP nuclear translocation, which in turn reinforces EZH2 expression, forming a vicious cycle that amplifies fibrotic signaling (122). Concurrently, mechanical stress induces nuclear deformation, directly increasing chromatin accessibility at mechanosensitive enhancers near genes such as ACTA2 and COL1A1 (123–125). These changes are further stabilized by stiffness-induced metabolic shifts, which alter the availability of metabolites such as α-ketoglutarate (α-KG) and S-adenosylmethionine (SAM), thereby influencing the activity of DNA methylation enzymes including DNMTs and TETs (126).

This mechanical reprogramming extends to immune cells within the fibrotic niche. In macrophages, substrate stiffness triggers a cytoskeleton–Src–p300 axis that drives histone H3 acetylation (H3Ac) at promoters of pro-inflammatory genes (e.g., IL1B, TNF), reinforcing M1-like polarization (77). The ensuing secretion of IL-1β and TNF-α not only amplifies inflammation but also directly activates surrounding fibroblasts, creating a feedforward loop. Evidence also suggests that mechanical cues, in concert with epigenetic regulators such as specific non-coding RNAs, may promote monocyte transdifferentiation toward a myofibroblast-like phenotype, directly expanding the pool of matrix-producing cells (127). In T cells, mechanical forces during T-cell receptor engagement can induce epigenetic modifications at cytokine loci, skewing the balance toward a pro-fibrotic Th17 response over a regulatory Treg phenotype, thereby sustaining chronic inflammation (128).

**Figure 5:**
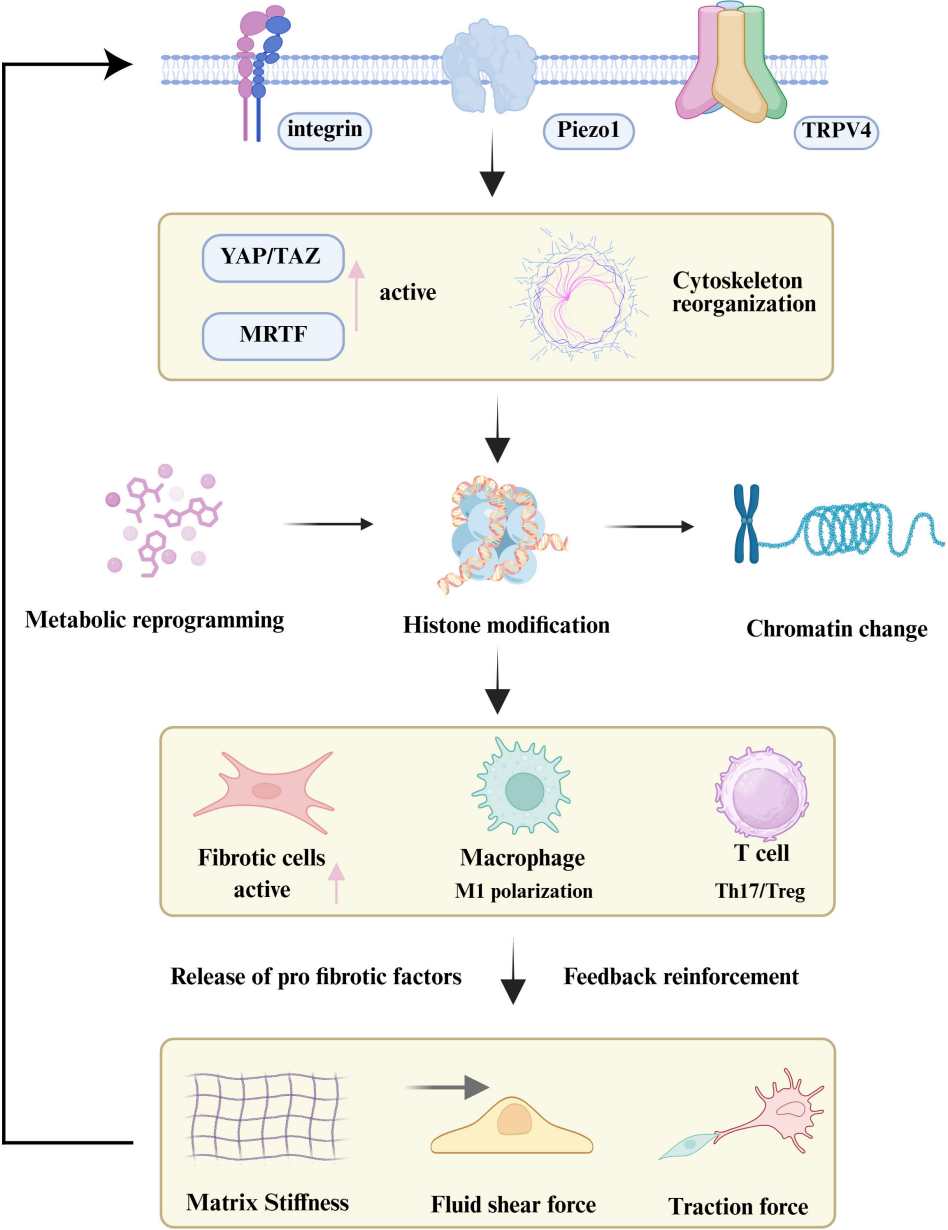
The mechano-epigenetic-immune axis in organ fibrosis. Persistent mechanical stimuli are sensed by cellular mechanoreceptors (e.g., Integrins, Piezo1), activating downstream signaling (YAP/TAZ) and inducing cytoskeletal reorganization. This force transmission leads to nuclear remodeling and widespread epigenetic reprogramming, which locks both stromal and immune cells into profibrotic activated states. The resulting secretion of factors creates a positive feedback loop that reinforces the fibrotic microenvironment, perpetuating disease progression.

In summary, epigenetic regulation stabilizes the persistent cellular activation that characterizes fibrosis. The mechano-immune axis, driven by these epigenetic programs, ensures that mechanical insults evolve into a sustained pathological dialogue between stromal and immune cells. Future studies should focus on dissecting cell-type-specific epigenetic codes and developing strategies to disrupt this dialogue and reverse the pathological “mechanical memory” for true disease regression. A schematic summarizing these epigenetic mechanisms within the mechano-immune axis is proposed in **Figure 5**.

## 4 Emerging therapeutic strategies targeting mechanotransduction-immune interactions in fibrosis

Traditional antifibrotic therapies have primarily focused on controlling inflammation, inhibiting fibroblast activation, and blocking collagen synthesis. However, their clinical efficacy has often been limited. In recent years, as understanding of fibrotic pathogenesis has deepened-particularly with the elucidation of the intersecting pathways of mechanotransduction and immune regulation-targeting the “mechanotransduction–immune axis” has emerged as a promising novel intervention strategy. This axis encompasses the entire cascade from mechanical stimulus sensing and intracellular signal transduction to immune cell activation and functional reprogramming, thereby providing a theoretical foundation and practical potential for multi-target synergistic therapies.

### 4.1 Signal pathway interventions targeting the mechanotransduction-immune axis

#### 4.1.1 Integrin signaling pathway

In fibrotic tissues, integrin expression is markedly upregulated, especially among the αv integrin family such as αvβ1, αvβ3, αvβ6, which can sense matrix stiffness and activate TGF-β, thereby inducing immune cell chemotaxis and fibroblast activation. By disrupting mechanical force transmission between cells and the ECM, integrin inhibitors not only attenuate TGF-β activation but also modulate immune cell adhesion and migration, effectively dampening pro-fibrotic inflammation at its source (129). For example, Cilengitide, an αvβ3/β5 antagonist, blocks immune cell adhesion and migration,inhibiting TGF-β–dependent myofibroblast differentiation, significantly reducing inflammatory infiltration and collagen deposition in liver and lung fibrosis models (130, 131). The αvβ6 antagonist Bexotegrast (PLN-74809) has advanced into Phase IIb/III clinical trials for idiopathic pulmonary fibrosis (IPF). Its mechanism involves specifically interrupting epithelial cell-derived TGF-β activation by the αvβ6 integrin, thereby disrupting the αvβ6/TGF-β positive feedback loop driving fibrosis progression (132). As primary mechanosensors at the “mechanotransduction-immune-fibrosis” axis, integrins represent promising targets for early intervention strategies.

#### 4.1.2 Mechanosensitive ion channels

Mechanosensitive ion channels are highly responsive to mechanical cues and constitute critical nodes linking biomechanical stimuli to immune regulation. Piezo1 inhibitors such as GsMTx4 suppress shear-induced macrophage M1 polarization and fibroblast activation in animal models (133). TRPV4 channel activation leads to calcium influx, which subsequently activates MAPK and NF-κB pathways to drive myofibroblast differentiation and ECM accumulation (134). TRPV4 antagonists, such as GSK2193874, have demonstrated potent antifibrotic effects in preclinical bleomycin-induced lung fibrosis models, significantly reducing IL-6 and collagen expression and lowering inflammation scores (135). Furthermore, TRPV4 activation contributes to ROCK pathway activation. This promotes cytoskeletal remodeling, increases cellular tension, and enhances fibroblast migration, thereby establishing the biomechanical foundation for fibroblast recruitment and activation (134). As key molecular integrators of mechanotransduction and immune/inflammatory signaling, TRPV4 channels represent compelling therapeutic targets for fibrosis.

#### 4.1.3 Rho/ROCK signaling pathway

Y-27632, a classical ROCK inhibitor, effectively suppresses TGF-β-driven fibroblast activation, attenuates myofibroblast differentiation and collagen deposition. For example, Y-27632 has been shown to prevent dimethylnitrosamine-induced hepatic fibrosis in rats (136). Additionally, several studies suggest that Y-27632 may influence macrophage polarization and dampen inflammatory responses (137, 138). Next-generation, highly selective ROCK2 inhibitors such as KD025 have demonstrated the ability to modulate STAT3 signaling,correct immune dysregulation, and suppress immune-mediated fibrotic progression (139–141).

#### 4.1.4 YAP/TAZ signaling

Targeting the YAP/TAZ signaling axis has emerged as a promising antifibrotic strategy. In a rat bleomycin-induced pulmonary fibrosis model, Zeyada et al. reported that trigonelline significantly inhibited YAP activity, evidenced by reduced nuclear translocation and downregulation of multiple pro-fibrotic genes (142). Haak et al. demonstrated that dihydrexidine (DHX), by activating dopamine D1 receptors, selectively suppresses YAP/TAZ activity, attenuating the profibrotic activation of pulmonary fibroblasts and hepatic stellate cells. In mouse models of pulmonary and cholestatic liver fibrosis, DHX treatment substantially attenuated collagen deposition and inflammation while preserving epithelial regenerative capacity (143). Statins, including simvastatin, have also shown antifibrotic potential in preclinical studies by promoting YAP cytoplasmic retention and inactivation, offering opportunities for drug repurposing (144, 145). Verteporfin, a direct inhibitor of YAP-TEAD binding and transcriptional activity, significantly reduced collagen I and fibronectin expression and ameliorated fibrosis severity in a unilateral ureteral obstruction (UUO) kidney fibrosis model, while improving epithelial structural integrity (146, 147). Emerging evidence also suggests that YAP/TAZ signaling modulates macrophage polarization and cytokine production, thereby linking mechanical cues to immune-driven fibrosis (148).

### 4.2 Smart delivery systems and responsive biomaterials targeting the mechanotransduction-immune axis

Achieving precise interventions targeting the mechanotransduction–immune axis requires overcoming the limitations of conventional pharmacotherapy, such as widespread biodistribution, limited specificity, and dose-limiting toxicity. In recent years, intelligent delivery platforms and engineered biomaterials have demonstrated significant advantages in controlling tissue specificity, cellular selectivity, and microenvironmental responsiveness (149–152). They are emerging as critical bridges linking mechanical signal modulation, immune regulation, and antifibrotic therapy. Particularly in fibrotic tissues characterized by pronounced aberrant mechanical cues, the development of nanocarriers with mechano-responsive properties has become a frontier in precision drug delivery. For example, shear stress–sensitive liposomes and polymeric nanoparticles can release anti-inflammatory or antifibrotic agents specifically at sites with elevated hemodynamic shear stress, enabling localized drug release triggered by mechanical forces (153, 154). In parallel, stiffness-sensitive nanocarriers selectively recognize regions of increased matrix rigidity and achieve spatial targeting through microenvironmental mechanical signals. This “mechanically responsive release” mechanism significantly enhances drug accumulation and therapeutic efficacy within fibrotic lesions (155).

The drug-loading capability of such materials further reinforces their role in targeted interventions. Nanocarriers can leverage the enhanced permeability and retention (EPR) effect in highly vascularized fibrotic foci, while active targeting ligands (e.g., peptides, antibodies) further enhance cellular specificity (156). For instance, mannose-modified albumin nanoparticles delivering TGF-β1 siRNA have been shown to effectively attenuate pulmonary fibrosis severity in murine models (157). In regenerative applications, bioengineered scaffolds can be combined with healthy cells or organoids to form implantable constructs that provide adhesive substrates and support functional tissue replacement. Proof-of-concept studies demonstrate that decellularized splenic matrix (DSM) can be repurposed to engineer 3D hepatic constructs with metabolic activity (158).

### 4.3 Advances in gene and cell engineering for mechanotransduction-immune precision therapy in liver fibrosis

Synthetic biology and gene engineering technologies are pioneering cell- and gene-level interventions to precisely target the mechanotransduction–immune axis in liver fibrosis. This disease exhibits high spatial heterogeneity and a dynamically evolving microenvironment where conventional small-molecule therapies often lack cellular specificity and sustained efficacy. Gene editing tools such as CRISPR/Cas9 now enable cell type-specific modulation of mechanosensitive pathways. For example, macrophage-specific knockout of Piezo1 or TRPV4 significantly reduces proinflammatory cytokine production and M1 polarization, thereby alleviating the persistent inflammatory stimulation of HSCs (159–161). Similarly, silencing of downstream mechanotransduction effectors like YAP/TAZ effectively suppresses ECM synthesis and abnormal immune activation (76). Animal studies employing lipid nanoparticles or viral vectors to deliver such gene-editing tools have demonstrated localized hepatic expression, low immunogenicity, and promising antifibrotic effects.

In terms of cell engineering, immune cells such as macrophages and T cells have been bioengineered to integrate both mechanosensory and immunoregulatory functions, broadening the potential of cell-based therapies in fibrosis. For instance, chimeric antigen receptor (CAR)-T cell technology, originally developed for cancer immunotherapy, is now being explored in fibrotic diseases. CAR-T cells designed to recognize fibroblast activation protein (FAP) on pathogenic fibroblasts and engineered to modulate mechanical signal transduction pathways may achieve selective clearance of fibrogenic cells while adapting to the altered mechanical microenvironment (162, 163).

### 4.4 Clinical challenges in mechanotransduction-targeted antifibrotic therapies

Despite the promising antifibrotic potential of targeting the mechanotransduction-mmune axis in animal models and early-phase clinical studies, the translation of these findings into clinical applications faces multiple challenges. For example, Bexotegrast has demonstrated good safety and favorable phase II outcomes in IPF, including attenuation of lung function decline, reduction of collagen metabolism biomarkers, and improvement in radiographic fibrosis scores. However, its ability to improve clinically meaningful endpoints such as overall survival remains to be validated in phase III trials. Furthermore, although receptor occupancy studies have confirmed Bexotegrast’s dose-dependent binding activity to αvβ6 integrin, clinical efficacy data are still lacking (164). The repositioning of integrin antagonists such as Cilengitide for fibrotic diseases has also encountered setbacks. In liver fibrosis models (e.g., TAA- or BDL-induced), Cilengitide unexpectedly exacerbated fibrotic progression, as evidenced by increased collagen septa thickness, upregulation of profibrotic genes TGF-β1 and TIMP-1/2 (165). These results underscore critical pharmacological limitations of Cilengitide—particularly suboptimal dosing regimens, lack of organ-specific targeting, and potential off-target profibrotic effects—which collectively hinder its repurposing for antifibrotic therapy. ROCK inhibitors such as KD025 offer a promising example of mechanotransduction-targeted therapies achieving regulatory approval for chronic graft versus host disease (GVHD), yet their long-term safety and efficacy in fibrotic diseases remain uncertain (166).

**Figure 6:**
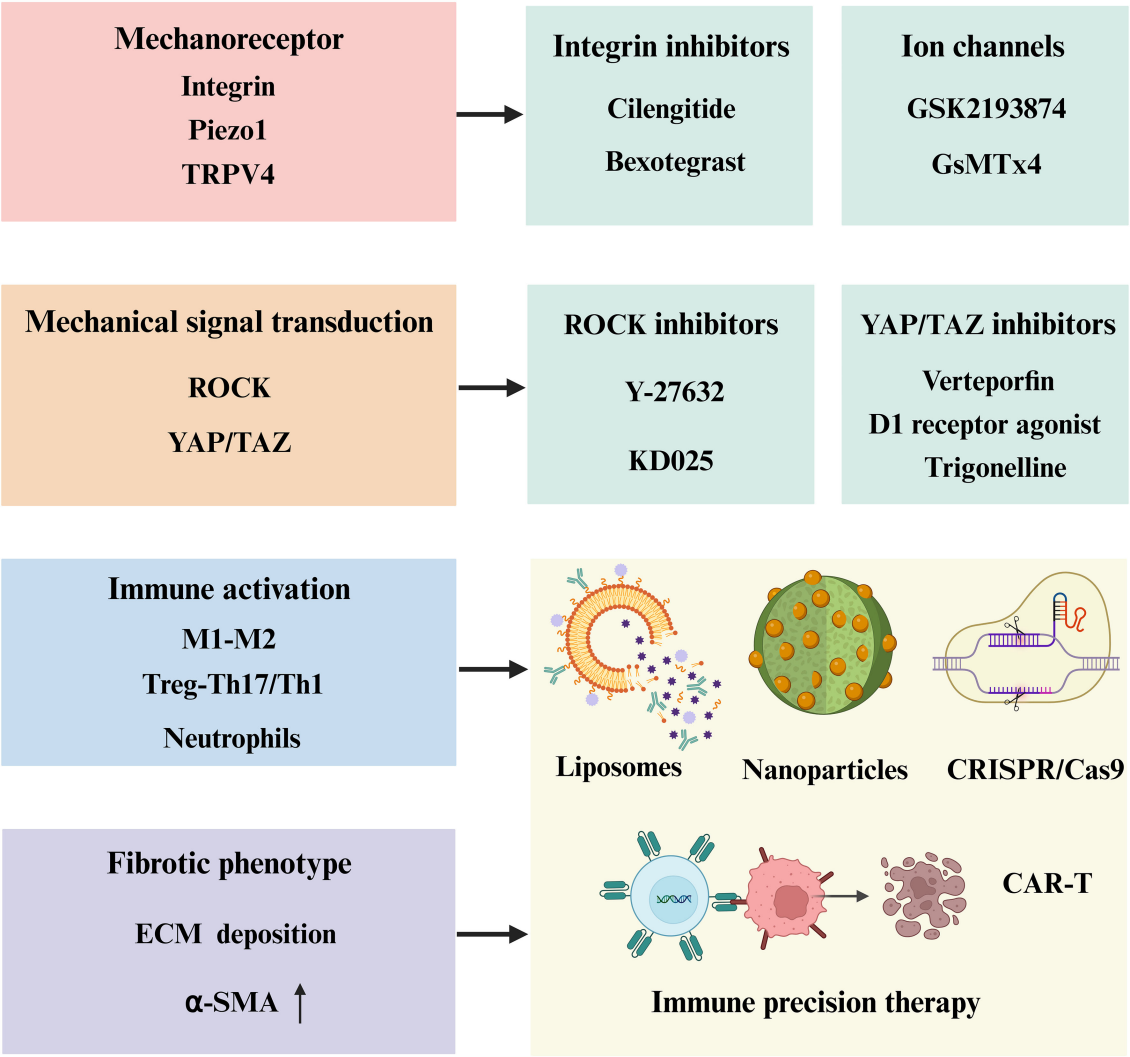
Emerging therapeutic approaches targeting fibrosis-related mechanotransduction and immune responses. Key strategies include inhibition of mechanoreceptors (Cilengitide [integrin inhibitor], Bexotegrast [integrin modulator], GSK2193874 [TRPV4 antagonist], GsMTx4 [Piezo1 inhibitor]), blockade of mechanical signaling (Y-27632, KD025 [ROCK inhibitor], verteporfin, dihydrexidine, trigonelline [YAP/TAZ inhibitor]), and modulation of immune activation via liposomes, nanoparticles, CRISPR/Cas9 editing, and CAR-T cell therapy to attenuate ECM deposition and myofibroblast activation.

In addition, organ-specific pathological and physiological differences pose major barriers to cross-organ translation. In liver fibrosis, key challenges include low drug delivery efficiency, widespread intrahepatic distribution, and potential target-related hepatotoxicity. In kidney fibrosis, high perfusion rates and steep pressure gradients complicate drug pharmacokinetics and targeting specificity. Other organs such as the lung, heart, and skin present additional barriers, including structural heterogeneity and distinct cellular targets. A common challenge across organs is the lack of strategies for precise delivery to fibrotic lesions. The multifocal, multi-organ distribution and heterogeneity of fibrotic foci remain major obstacles to efficient and specific targeting. Although advanced delivery platforms such as nanocarriers and gene therapies have emerged, most remain at the preclinical stage and face translational and safety concerns. Moreover, the field lacks standardized, clinically relevant endpoints. Current studies rely heavily on surrogate biomarkers, with insufficient emphasis on long-term outcomes such as survival and organ function. The translational gap between animal models and human disease is also substantial, particularly in terms of tissue stiffness, chronic inflammation, and microenvironmental complexity, all of which limit the predictive value of preclinical results. **Figure 6** and **Table 1** present emerging antifibrotic therapies that target mechanotransductive and immune pathways, alongside key translational barriers and representative agents under investigation across fibrotic organ systems.

**Table 1:** Translational challenges and representative therapies targeting the mechanotransduction-immune axis in organ fibrosis.

Organ/System	Representative targets/Therapies	Clinical development stage	Translational challenges
Pulmonary Fibrosis (130–132, 135, 143, 157, 164–166)	αvβ6/αvβ1 integrin inhibitors (Bexotegrast); ROCK2 inhibitor (KD025); TRPV4 antagonist (GSK2193874); αvβ3/β5 antagonist (Cilengitide); D1 receptor agonist (Dihydrexidine). TGF-β1 siRNA in mannose-modified albumin nanoparticles.	Bexotegrast: Phase IIb/III; KD025: under fibrosis; TRPV4 inhibitors: Preclinical; D1 agonists: Preclinical; siRNA nanotherapy: Preclinical.	Disease heterogeneity complicates endpoint selection Need for long-term efficacy data Safety concerns with existing antifibrotics.
Liver Fibrosis (76, 136, 143, 159–161)	ROCK inhibitor (Y-27632); D1 receptor agonist (Dihydrexidine); Macrophage-specific Piezo1/TRPV4 knockdown; YAP/TAZ inhibition via LNPs or viral vectors.	D1 agonists: Preclinical; Y-27632/D1 agonists: Preclinical.	Intrahepatic delivery is hindered by complex hemodynamics; Risk of hepatotoxicity in multi-target strategies.
Renal Fibrosis (146, 147)	YAP/TAZ inhibition (verteporfin).	Verteporfin: Preclinical.	Renal perfusion and glomerular pressure alter pharmacokinetics; Limited success in translating rodent models to humans.

## 5 Conclusion and perspectives

Organ fibrosis is increasingly recognized not merely as the end result of immune overactivation or chronic inflammation, but as a dynamic pathophysiological process governed by biomechanical regulation and immunological reprogramming. The evolving fibrotic microenvironment generates sustained mechanical cues—including matrix stiffening, interstitial shear stress, and architectural distortion—that not only activate fibroblasts but also reshape immune cell behavior, differentiation, and metabolism. In this context, the mechanotransduction–immune axis has emerged as a unifying framework integrating physical and immunological inputs into a self-reinforcing fibrotic circuit. Based on this understanding, a conceptual stage-specific model can be envisioned, aligning mechanical and immune processes with fibrosis evolution. In the early stage, tissue injury triggers damage associated molecular patterns (DAMP)-mediated immune priming and initial fibroblast recruitment. Concurrent subtle ECM remodeling begins to engage mechanosensors like Piezo1, TRPV4, and integrins. Interventions during this phase may benefit from combined targeting of immune polarization and mechanosensors, as suggested by recent findings (159, 161). During the amplification phase, mechanotransductive signals converge with inflammatory pathways, YAP/TAZ activation synergizes with TGF-β signaling to promote myofibroblast transition and immune polarization (167–169). This stage is characterized by a pathological “mechanical–immune–fibroblast” positive feedback loop. In the late stage, extensive ECM crosslinking induces irreversible stiffening and “mechanical memory,” sustaining α-SMA myofibroblast activation and impeding immunomodulation and tissue repair (168). Effective treatments must reverse stiff ECM and eliminate fibrogenic cells, while minimizing collateral tissue damage. Such a stage-target-mechanism paradigm may offer a framework for precision interventions across fibrotic diseases. Viewing fibrosis as an adaptive, rhythm-dependent, mechanobiological network underscores the necessity of time-adaptive mechanism-guided interventions for true reversal. **Table 2** provides a comparative summary of current and emerging anti-fibrotic therapies, organized by molecular targets, mechanisms of action, applicable fibrosis stages, and translational readiness, offering a concise reference for researchers and clinicians.

**Table 2:** Comparative overview of fibrosis-targeted therapeutic strategies: molecular targets, mechanisms of action, fibrosis stage applicability, and translational readiness.

Target/Strategy	Molecular targets/Therapeutic platforms	Mechanism of action	Fibrosis stage	Translational readiness
Integrin inhibitors	Molecular targets	Block latent TGF-β activation; reduce fibroblast activation and immune adhesion	Early-mid stage (suppresses initiation of myofibroblast activation)	αvβ6 inhibitor (Bexotegrast) in Phase IIb/III trials (IPF); others in preclinical/early clinical
Ion channel modulators		Inhibit mechanosensitive calcium influx; modulate macrophage polarization and cytokine release	Early-progressive stage (controls inflammatory amplification)	Selective inhibitor GsMTx4 tested preclinically; TRPV4 antagonist (GSK2193874) shows preclinical antifibrotic efficacy
ROCK inhibitors		Block RhoA-ROCK signaling; reduce cytoskeletal tension	Amplification (mechanical-immune positive feedback)	ROCK inhibitors (Y-27632, KD025) in preclinical; KD025 approved for GVHD, under fibrosis evaluation
YAP/TAZ inhibitors		Prevent YAP/TAZ nuclear translocation; inhibit transcription of profibrotic genes	Amplification-Late (myofibroblast transition, immune reprogramming)	Verteporfin preclinical; Statins repurposing; DHX preclinical; trigonelline preclinical
Macrophage reprogramming		Shift macrophages from M1 to M2 or regulatory phenotypes	Early - Late (immune priming, fibrosis amplification, matrix stiffening)	Preclinical: Piezo1/TRPV4 KO; ROCK2 inhibitors reduce macrophage contractility
T cell modulation		Control Treg/Th17 balance via mechanical cues	Amplification (inflammation-fibrosis loops)	Preclinical
Neutrophil targeting		Block extracellular trap formation	Early-mid stage	Preclinical
Nanomedicine & biomaterials	Therapeutic platforms	Stiffness-/shear-responsive delivery; targeted siRNA, drug release	Multiple stages (context-specific)	Preclinical development
Gene editing (CRISPR/Cas9)		Knockout mechanosensors in immune/stromal cells	Progressive-late stage	Proof-of-concept, preclinical
Cell therapies (FAP-CAR-T, engineered macrophages/T cells)		Eliminate activated myofibroblasts; reprogram immune responses	Progressive-late stage	Early preclinical

Future work should focus on defining immune-subtype-specific mechanosensing mechanisms, engineering adaptive therapeutics responsive to mechano-immune signals, and developing mechanobiological biomarkers that integrate tissue stiffness imaging, interstitial flow dynamics, and immune cell mechanosensitivity thresholds. In this emerging paradigm, fibrosis is not a static scarring process, but a dynamic mechano-immune dialogue with encoded structural and informational memory, which is ultimately interceptable through biomechanically-tuned interventions. Such an approach promises to transform fibrotic disease management into one that is precise, plastic, and cross-organally applicable.
